# Using a mixed‐methods approach to adapt an HIV stigma reduction to address intersectional stigma faced by men who have sex with men in Ghana

**DOI:** 10.1002/jia2.25908

**Published:** 2022-07-12

**Authors:** Laura Nyblade, Melissa A. Stockton, Khalida Saalim, Gamji Rabiu Abu‐Ba'are, Sue Clay, Mutale Chonta, Debbie Dada, Emmanuel Mankattah, Richard Vormawor, Patrick Appiah, Francis Boakye, Ransford Akrong, Adom Manu, Emma Gyamerah, DeAnne Turner, Karan Sharma, Kwasi Torpey, LaRon E. Nelson

**Affiliations:** ^1^ Global Health Division International Development Group RTI International Washington DC USA; ^2^ Department of Psychiatry Columbia University Irving Medical Center New York New York USA; ^3^ Center for Interdisciplinary Research on AIDS School of Public Health Yale University New Haven Connecticut USA; ^4^ 3C Regional Consultants Zambia Africa; ^5^ School of Nursing Yale University New Haven Connecticut USA; ^6^ Educational Assessment & Research Center Accra Ghana; ^7^ Youth Alliance for Health & Rights Kumasi Ghana; ^8^ Priorities on Rights & Sexual Health Accra Ghana; ^9^ Department of Population Family & Reproductive Health School of Public Health University of Ghana Legon‐Accra Ghana; ^10^ College of Nursing University of South Florida Tampa Florida USA; ^11^ Factor‐Inwentash Faculty of Social Work University of Toronto Toronto Ontario Canada; ^12^ Yale Institute for Global Health School of Public Health New Haven Connecticut USA

**Keywords:** stigma, intervention, men who have sex with men, Africa, HIV care continuum, key and vulnerable populations

## Abstract

**Introduction:**

In Ghana, men who have sex with men (MSM) are estimated to be 11 times more likely to be living with HIV than the general population. Stigmas at the intersection of HIV, same‐sex and gender non‐conformity are potential key drivers behind this outsized HIV disease burden. Healthcare workers (HCWs) are essential to HIV prevention, care and treatment and can also be sources of stigma for people living with HIV and MSM. This article describes the process and results of adapting an evidence‐based HIV stigma‐reduction HCW training curriculum to address HIV, same‐sex and gender non‐conformity stigma among HCWs in the Greater Accra and Ashanti regions, Ghana.

**Methods:**

Six steps were implemented from March 2020 to September 2021: formative research (in‐depth interviews with stigma‐reduction trainers [*n* = 8] and MSM living with HIV [*n* = 10], and focus group discussions with HCWs [*n* = 8] and MSM [*n* = 8]); rapid data analysis to inform a first‐draft adapted curriculum; a stakeholder adaptation workshop; triangulation of adaptation with HCW baseline survey data (*N* = 200) and deeper analysis of formative data; iterative discussions with partner organizations for further refinement; external expert review; and final adaptation with the teams of HCWs and MSM being trained to deliver the curriculum.

**Results:**

Key themes emerging under four immediately actionable drivers of health facility intersectional stigma (awareness, fear, attitudes and facility environment) informed the adaptation of the HIV training curriculum. Based on the findings, existing curriculum exercises were placed in one of four categories: (1) Expand—existing exercises that needed modifications to incorporate deeper MSM and gender non‐conformity stigma content; (2) Generate—new exercises to fill gaps; (3) Maintain—exercises to keep with no modifications; and (4) Eliminate—exercises that could be dropped given training time constraints. New exercises were developed to address gender norms, the belief that being MSM is a mental illness and stigmatizing attitudes towards MSM.

**Conclusions:**

Getting to the “heart of stigma” requires understanding and responding to both HIV and other intersecting stigma targeting sexual and gender diversity. Findings from this study can inform health facility stigma reduction programming not only for MSM, but also for other populations affected by HIV‐related and intersectional stigma in Ghana and beyond.

## 1 INTRODUCTION

Recognizing the role of stigma in the health and wellbeing of people living with, at risk of, or affected by HIV, the UN General Assembly political declaration on HIV and AIDS established the target of reducing stigma and discrimination to 10% by 2025 [1]. Efforts must now expand stigma‐reduction programmes and build the evidence for effective implementation. “To get to the heart of stigma,” these efforts must focus on key populations (KPs) affected by HIV and healthcare workers (HCWs) [2]. Their importance is underlined by specific 2025 global stigma sub‐targets: less than 10% of HCWs will report stigmatizing attitudes towards KPs or people living with HIV (PLHIV) and less than 10% of KPs will experience stigma [2].

Ensuring KPs, including gay, bisexual and other men who have sex with men (MSM), have access to stigma‐free HIV prevention and treatment services is a human rights imperative and key to ending AIDS by 2030 [3]. Between 2010 and 2019, MSM experienced a 25% increase in HIV infections [2]. Stigma at the intersection of HIV, same‐sex and gender non‐conformity has been identified as a potential key driver behind the outsized HIV disease burden among MSM [4, 5, 6]. Intersectional stigma occurs at the juncture of multiple stigmatized identities, arises from systems of oppression and may be synergistic in effect [7, 8, 9, 10]. The stigma experienced by MSM is especially acute in countries where the legal, social and cultural milieu forces many MSM underground [11, 12, 13, 14, 15], making accessing health services, including HIV services, challenging [16, 17]. In many West African countries, including Ghana, stigma manifests through laws criminalizing homosexuality, impeding HIV prevention and treatment services for MSM [15, 18, 19]. While there is no specific Ghanaian law denouncing MSM, section 104 of the Criminal Code, “Unnatural carnal knowledge is sexual intercourse with a person in an unnatural manner or with an animal” is commonly interpreted to include same‐sex behaviour [17, 20].

HCWs and healthcare facilities (HCFs) are essential to HIV prevention, care and treatment. They can also be sources of stigma for both PLHIV and KPs [21, 22], who have reported dismissive attitudes, coerced procedures and refusal to deliver treatment [2, 6, 23, 24]. In an HCF study in Ghana, 29% of HCWs indicated that if given the choice, they would prefer not to provide services to MSM [6]_._ The critical role of HCFs and HCWs in tackling stigma is emphasized by UN‐led global HCF stigma‐reduction initiatives [25, 26, 27]. Multiple HIV HCF stigma‐reduction interventions are available [21, 22, 24, 28, 29, 30, 31, 32], and a few studies have developed interventions to address MSM stigma [33, 34, 35]. However, interventions to address intersectional (HIV, same‐sex and gender non‐conformity) stigma faced by MSM, particularly in West Africa, are limited [9, 23, 36].

In response, this article describes the process of adapting an evidence‐based HIV stigma‐reduction training curriculum, the Health Policy Plus (HP+) total facility approach [29], to address intersectional HCF stigma towards MSM in Ghana. This adaptation was conducted as an initial step in a study that is testing multi‐level, intersectional stigma‐reduction interventions to address HIV, same‐sex and gender non‐conformity stigma towards and among MSM in eight communities and HCFs in the Greater Accra and Ashanti regions, Ghana [37]. The latter region is more homogenous (most residents are Ashanti) and closely interconnected, whereas the former is more cosmopolitan and “anonymous,” and the traditional setting of the Ga may be more accepting of non‐conforming gender expression. However, within HCFs in both regions, HCWs come from across the country. Guided by the social ecological [38, 39] and ADAPT‐ITT models [40], this study is adapting, integrating and testing evidence‐based HIV stigma‐reduction interventions at the organizational (HCF) [29], interpersonal (among MSM communities) [41] and intrapersonal (within the individual) [42] levels to address intersectional stigma towards and among MSM [Clinical Trials Registration #:NCT04108078]. This manuscript describes the adaptation process for the HCF‐level intervention [43].

The HP+ HIV stigma‐reduction “Total Facility” approach [29] targets the whole HCF, recognizing that stigma can occur in client interactions with both clinical and non‐clinical HCWs and in HCF institutional processes and structures. It includes three phases—formative research, HCW and client capacity building through participatory training workshops and integration of stigma‐reduction into HCF structures and processes. It targets four immediately actionable drivers of HIV stigma: (1) awareness and understanding of how stigma manifests in daily lives and interactions; (2) fear of HIV acquisition in routine contact with PLHIV; (3) attitudes which lead to shaming, blaming, judgement and stereotyping; and (4) institutional environment, structures, policies and practices that either sustain or reduce stigma [3]. HCWs and clients receive a 5‐day training and a week of on‐site coaching as they work as teams to jointly deliver participatory stigma‐reduction training sessions in their facilities [43, 44, 45]. Each session includes a mix of up to 30 clinical and non‐clinical staff across departments. Modular sessions accommodate differing hospital schedules. Depending on the setting and adaptation, the curriculum can range from a total of 6–14 hours delivered in 1–3 hour sessions [46, 47]. Stigma‐reduction champions, which emerge organically from the trainings and are supported by HCF management, develop and implement additional stigma‐reduction activities in their facilities. Such activities include onboarding new staff and incorporating stigma‐reduction into existing practices, like rounds, staff recognition and complaint/compliment systems. In Ghana, the HP+ curriculum included one module focused on building understanding of sexual and gender diversity [44, 48].

## 2 METHODS

**Figure 1:**
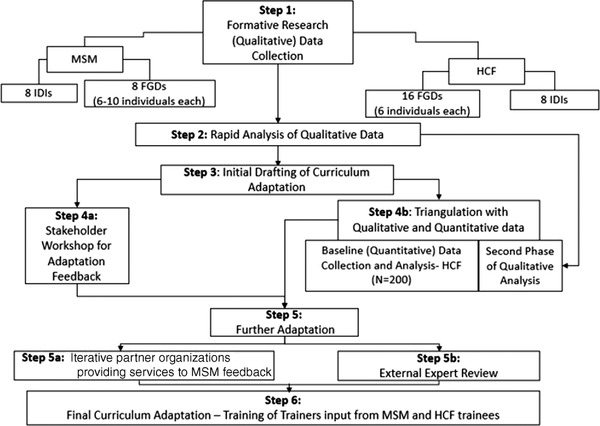
Curriculum adaptation methods and process. Abbreviations: FGDs, focus group discussions; HCF, health care facilities; IDIs, in‐depth interviews; MSM, men who have sex with men.

The process of adapting the HP+ HIV stigma‐reduction curriculum to address HCF intersectional MSM stigma included: formative research with rapid data analysis to inform a first‐draft adapted curriculum; a stakeholder adaptation workshop; triangulation of adaptation with baseline HCW data and deeper analysis of formative data; iterative discussions with partner organizations providing services to MSM for further refinement; external expert review; and final adaptation with the teams of HCWs and community members being trained to deliver the curriculum (Figure 1). These activities were conducted from March 2020 to September 2021.

### 2.1 Formative research

#### 2.1.1 Population and sampling

##### HCF

Eight In‐depth interviews (IDIs) were conducted with a convenience sample of HP+ HCF trainers invited to participate by telephone, with interviews conducted over Zoom. Sixteen Focus group discussions (FGDs) (six participants each) were conducted in person with convenience samples of clinical or non‐clinical HCF staff employed at an HCF participating in the parent study. Interviewers worked with HCF management the day of the FGDs to identify separate groups of clinical and non‐clinical staff and invite available staff to participate.

##### MSM

Eight FGDs (6–10 participants each) were conducted through snowball sampling [49] of adult MSM (≥18 years) who were assigned male sex at birth, identified as cisgender men and reported sex with another man within the previous 6 months. MSM members of study partner organizations who offer MSM health and advocacy services identified eligible participants in their networks (either in‐person or by telephone) and invited them to attend scheduled FGDs. No socio‐demographic or health information was collected from FGD participants, and we did not endeavour to diversify the FGDs, limiting the generalizability to Ghana's entire MSM population. To protect confidentiality of HIV status, IDIs (*n* = 8) were conducted only with MSM who self‐disclosed they were living with HIV and met the FGD eligibility criteria. To protect MSM respondents, no personal identifying information was collected, apart from signatures on consent forms kept in a locked cabinet. Keeping confidentiality was discussed with FGD participants who used pseudonyms in the FGDs. Interviews were held at the partner organization offices, which are known safe spaces in the MSM community but identified in the wider community only as community health organizations.

#### 2.1.2 Data collection

We trained research assistants experienced in HIV research to conduct the HCF IDIs and FGDs and MSM identified through the partner organizations to conduct the MSM IDIs and FGDs. These interviewers conducted the IDIs (1 hour) and FGDs (1.5–2 hours) using semi‐structured guides in English or Twi, based on participants’ preferences, and recorded, transcribed and translated as necessary. COVID precautions were implemented including masking, social distancing and hand sanitizing.

#### 2.1.3 Data analysis

Data analysis used an iterative process that began with a “rapid analysis” to immediately inform the intervention adaption. The “rapid analysis” consisted of reviewing the transcripts and creating analytic summaries for each transcript [50]. The research team developed a summary template that captured: (1) MSM stigma drivers and manifestations; (2) HIV stigma drivers and manifestations; (3) intersectional stigma; (4) how stigma undermines HIV prevention and testing for MSM; (5) MSM‐friendly services; (6) stigma reduction; and (7) the “Total‐Facility” intervention. Seven team members reviewed the transcripts and used the template to take detailed notes on the emerging themes and record pertinent quotes. MS and GMRA collated these summaries into a singular “rapid‐analysis” document, which informed the initial draft of the curriculum adaptation and intervention stakeholder adaptation workshop.

A more robust thematic analysis followed the “rapid‐analysis.” After reviewing every transcript, MS performed inductive “open” coding of at least one of each type of transcript [51, 52]. MS worked with the research team to develop a thematic codebook to capture drivers and manifestations of HIV and MSM stigmas and suggestions to improve the training. Additionally, deductive codes were created using the existing Health Stigma and Discrimination Framework [53], to help structure the exploration of intersectional stigma, and the Consolidated Framework for Implementation Science Research [54], to guide efforts preparing for intervention implementation. A team of five coders individually applied the codebook to the same four transcripts, meeting after each transcript to review the line‐by‐line coding, discuss discrepancies, make changes to the codebook and ultimately ensure consistency in coding application. The remaining transcripts were coded by one of these five individuals. Data were managed, coded and analysed using Dedoose 8.3. Upon completion of coding, the research team executed queries in Dedoose and reviewed coded data relevant to the intervention adaptation.

### 2.2 Quantitative research

HCF baseline data were utilized to triangulate the formative findings to confirm and further refine the adaptation. The main study is ongoing, and future data collection rounds will allow for assessment of the full HCF intervention, including the training and additional stigma‐reduction activities; this article focuses solely on the curriculum adaptation process.

#### 2.2.1 Population and sampling

Eligible HCF staff (*N* = 200) included both clinical and non‐clinical staff employed at a hospital in the parent study [37]. Purposive sampling recruited staff likely to interact with MSM clients by selecting 60% of the sample from key departments (Antiretroviral therapy (ART) clinic, outpatient department, pharmacy and security/reception/management) and 40% from other departments. On data collection days, staff arriving first in the designated departments were invited to participate until sample targets were reached.

#### 2.2.2 Data collection and analysis

Data were collected using self‐administered paper questionnaires, with interviewers present to assist as needed. All data were double‐entered, and any discrepancies checked against the paper surveys. We present frequencies of key variables and summary statistics relevant to the four actionable drivers and the intervention adaptation process.

### 2.3 Curriculum adaptation

Initial adaptation of the curriculum was done by two stigma‐reduction master trainers (SC and MC) and one principal investigator (LN), all of whom tailored the HP+ “Total Facility” approach HIV stigma‐reduction curriculum for Ghana [29, 44, 48]. The team reviewed the original HIV stigma‐reduction curriculum considering findings from both study regions in the “rapid analysis.” They identified the exercises that were: still relevant; relevant but needed additional intersectional stigma content; or irrelevant and could be dropped. As well, they noted where gaps existed that required new exercises. A participatory workshop with the full research team, which included MSM from partner organizations from each study region, was then held to discuss the “rapid analysis” findings and the initial curriculum adaptation. Based on these deliberations, the team adapted the existing exercises and created new ones, with support from both MSM partner organizations. An external stigma‐reduction expert trainer then reviewed the adapted curriculum and further revisions ensued. Final adaptations occurred during the 5‐day training‐of‐trainers of HCWs and MSM from both regions, who were to deliver the training in their respective facilities. HCF baseline data from both regions were utilized to triangulate and confirm the adaptation.

### 2.4 Ethics

We obtained ethics approval from Yale University, Noguchi Memorial Institute for Medical Research, University of Toronto and Ghana Health Services. All respondents provided written informed consent after undergoing an informed consent process, which provided study information and stressed that participation was voluntary and would not impact their HCF employment or services relationship with the partner organization.

## 3 RESULTS

The adaptation process led to existing curriculum exercises being placed in one of four categories: (1) Expand—modify existing exercises to incorporate or deepen MSM and gender non‐conformity stigma content; (2) Generate—create new exercises; (3) Maintain—keep exercises with no modifications; and (4) Eliminate—drop exercises given time constraints. The original Ghana HP+ curriculum [44, 55] had 14 exercises. In the adapted curriculum, eight of the original exercises were kept (four with no changes and four with added intersectional stigma content), three new exercises were created and six original exercises were dropped. We describe key themes that informed the above categorization, organized by four key immediately actionable drivers [30], and highlight HCW survey data that triangulated the theme. Table 1 summarizes the findings by key driver, corresponding curriculum topic and specific exercises in the adapted curriculum.

**Table 1:** Mixed‐methods data informing curriculum adaptation, by curriculum topic and training exercise

Immediately actionable driver	Qualitative findings (MSM)	Qualitative findings (HCF and HP+ trainers)	Quantitative findings (HCF)	Curriculum topic	Workshop exercise
Awareness and knowledge	X	X	X	1. Building understanding and awareness of what stigma looks like in concrete terms with a focus on HIV, MSM and gender non‐conforming stigma, gender norms and stigma, stigma, MSM and mental health	Naming stigma through pictures [EXPAND]
Attitudes	X	X	X	2. Building empathy and reducing distance	Values clarification [GENERATE]
					Outside the gender box [GENERATE]
					How myths about MSM and mental illness can lead to stigma [NEW]
					Identity soup [MAINTAIN]
					Gender and sexual diversity [EXPAND]
					Gender and sexual diversity terminologies [EXPAND]
					Listen to first‐hand experiences of people experiencing stigma; discuss experiences in health facilities [MAINTAIN]
					Self‐reflection [MAINTAIN]
Fear	N/A	X	X	Understanding and addressing fear of contracting HIV in the workplace	Fears about nonsexual transmission/quantity, quality and route of entry (QQR) [MAINTAIN]
Institutional environment	X		X	Understanding the importance of confidentiality and the link to stigma	Confidentiality and stigma [EXPAND]
			X	Building skills to address stigma and planning action to address stigma within health facilities	Challenge the stigma and be the change [EXPAND]
					Writing a code of practice and action plan [EXPAND]

Abbreviations: HCF, health care facilities; HP+, health policy plus; MSM, men who have sex with men.

### 3.1 Immediately actionable drivers

#### 3.1.1 Driver 1: Awareness

Three themes emerged relevant to awareness: (1) lack of recognition of how MSM stigma manifests in health facilities, (2) limited understanding of how gender norms undergird intersectional stigma faced by MSM and (3) the belief that being MSM is a mental illness.

1) HCW appeared not to recognize how their own stigmatizing attitudes and beliefs may manifest in service delivery to MSM (even if unconsciously). This was evident in the HCW FGDs through a disconnect between clear descriptions of strongly held stigmatizing attitudes and beliefs about MSM and stigmatizing behaviours—such as bringing religion into the patient–provider encounter in a manner that judges MSM—and repeated statements from HCWs that services are delivered to MSM without stigma.

*It depends on the whole situation. There are some people [MSM] you can easily convince them. For instance…I'll make sure I'm very close to you [MSM client]. I'll get to know what you really do. What really allowed you to be in there. I'll make sure I agree with you all the time. Then as time goes on, I try to convince you to come back… But there are some people it's very difficult to convince them. Very. You can read the bible from Genesis to Revelation, they don't hear. (FGD, Clinical HCW)*

**Figure 2:**
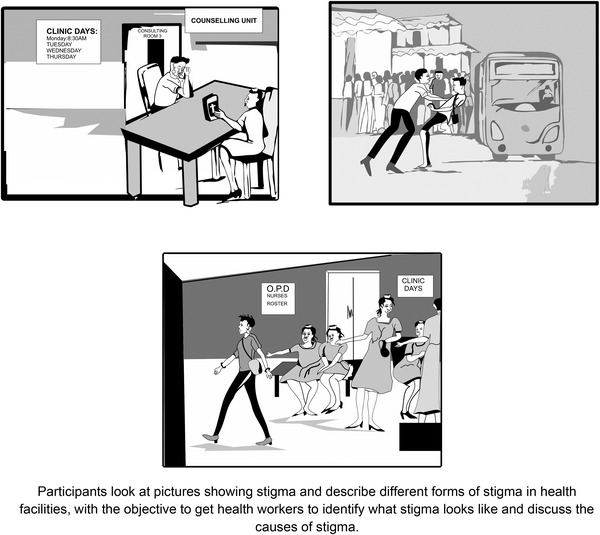
Sample of redrawn pictures depicting stigma towards MSM for use in the “Naming Stigma Through Pictures” exercise.

Several of the original HP+ curriculum exercises respond to this driver by building understanding and awareness of stigma in general (e.g. *Self‐Reflection* exercise). For the adaptation, the *stigma awareness* exercises were deepened by adding MSM and gender non‐conformity content. For example, in the *Naming Stigma through Pictures* exercise (Figure 2), the team reviewed existing pictures and determined if they should be redrawn to be more contextually appropriate for the two study regions and where new pictures were required.

2) The second theme under the awareness driver is the strong gender norms around how “real and proper” Ghanaian men and women should look, dress, behave and uphold “traditional” marriage and childbearing and rearing norms. Men and women ostensibly “should” get married (to the opposite gender) and have children, with men as head of the household and responsible for taking care of the family financially, while women raise the children and manage domestic affairs. Both the HCW and MSM data demonstrate how MSM and gender non‐conforming stigmas are shaped and driven by these strongly held traditional gender norms and a lack of awareness of the relationship between gender norms and MSM stigma:

*R1: For a Ghanaian man you are supposed to be responsible, pay the bills in the house…take care of your wife*.

*R2: Our culture makes us to understand that a man is a man. He should be in trousers, should walk masculine, have this masculine feature. He should behave like a boy, a man, that's what our culture tells us. (FGD, Clinical HCW)*

*My community is a bit hostile to Saso people [MSM], especially if you exhibit signs of femininity. They believe that as a man you have to behave like a man. I almost got killed because they think I am a curse or something to the community. They think I am not human and don't deserve to live. (FGD, MSM)*

Responses to several gender norms statements from the HCW baseline data mirror the qualitative gender norms findings (Table 2).

**Table 2:** Agreement with gender norm statements related to MSM and gender non‐conforming stigma among healthcare facility staff (*N* = 200)^a^

Do you strongly agree or disagree with the following statements?	Agree	Neither agree nor disagree	Disagree
A man should be able to dress like a woman, if he chooses.	8.2% (*N* = 195)	15.9% (*N* = 195)	75.9% (*N* = 195)
A woman should be able to present herself as a man in public, if she chooses.	11.6% (*N* = 198)	15.7% (*N* = 198)	72.7% (*N* = 198)
If a man has attraction/feelings for other men, they should do everything to overcome these feelings.	77.7% (*N* = 197)	13.2% (*N* = 197)	9.1% (*N* = 197)
If a person feels that they want to present their mannerisms, dress or practices in a different gender than the one they were born into (such as feminine presenting men), they should do everything to overcome these feelings.	62.6% (*N* = 198)	24.2% (*N* = 198)	13.1% (*N* = 198)

^a^ *N*’s may vary due to non‐response.

Abbreviation: MSM, men who have sex with men.

**Figure 3:**
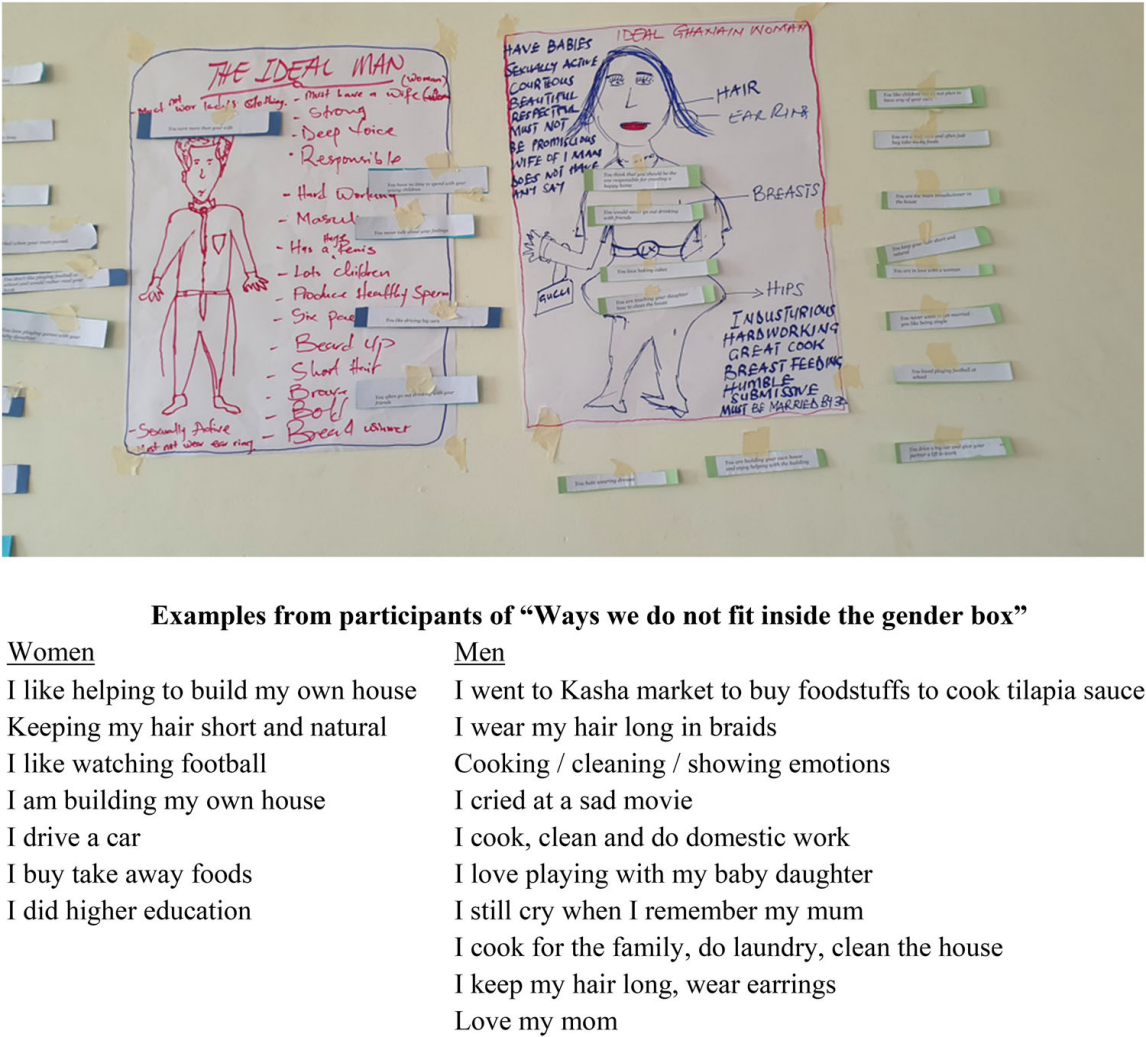
Output from the gender box exercise.

In response, we deepened the gender content in the curriculum by adding a new exercise and expanding the existing sexual and gender diversity exercise. These two exercises, along with an in‐person panel of MSM, work to create awareness of gender norms and how they relate to MSM stigma by building understanding of gender and sexual diversity and building empathy through in‐person “contact” with MSM outside of a clinical setting. The exercises also promote self‐reflection by trainees on how they express themselves, often on a daily basis, in ways that are outside of traditional gender norms or the gender role assigned to them at birth, and how that makes them feel. The new exercise on gender norms, O*utside the Gender Box*, was adapted from the *Keep the Best, Change the Rest* manual [56]. This exercise (Figure 3) was added to: explore societal gender norms and how they influence upbringing, attitudes and beliefs; examine the negative impact that gender norms can have on our lives and those who do not conform to gender norms; reflect on how we have stepped outside of gender norms in our own lives; and explore the link between gender norms and stigma, particularly towards gender non‐conforming people. It is an interactive participatory exercise that ends with a debrief discussion.

3) The third theme emerging under the awareness driver is the belief that being a MSM is a symptom of mental illness. This belief manifests in thinking that MSM should be referred for services to address their “MSM mental illness.” This belief appears to be reinforced by outdated medical training of same‐sex attraction as a psychiatric diagnosis:

*Such men [MSM], actually psychologically, they have a problem; if it is not addressed, okay psychologically they have a problem; they have to be looked at carefully or counseled to desist from that. (FGD, Clinical HCW)*

Quantitative data reinforced the importance of addressing the beliefs around MSM being a mental illness. Only a third (33.8%) of HCWs disagreed with the statement *Being MSM is a mental illness*, while over a third (37.4%) agreed and 28.7% neither agreed nor disagreed.

In response to this theme, a new exercise *How myths about MSM and mental illness can lead to stigma* was developed that combines a role play, short power point presentation and discussion. The objectives are to learn about research and beliefs around sexuality and mental disorders, discuss the myths that participants may have been taught and understand the link between stigma and mental health. A short presentation, developed by a senior Ghanaian psychiatrist and refined by a facility trainer, begins by first defining mental health and its relationship to physical, social and emotional wellbeing, in contrast to mental illness. Key myths and misconceptions are then named and debunked through facts. The consequences of these myths—such as how myths can drive stigma, rejection, social isolation and mental illness (e.g. depression)—are also explored. The session ends with discussion about how to change the situation.

#### 3.1.2 Driver 2: Fear

Fear as a driver of stigma was distinct for HIV and MSM stigmas. Given fear of acquiring HIV while providing care to PLHIV is already documented in Ghana as an important HIV stigma driver to address in health facilities [48], the qualitative guides did not focus on this issue. However, it is still surfaced in the qualitative discussions and the baseline survey confirmed that it remains an issue as 56.4% of HCWs expressed fear of HIV acquisition through at least one of four routine client care interactions, while 60.6% indicated they routinely use one of four unnecessary infection control measures when providing care for PLHIV. Further, 33.3% of HCWs thought their co‐workers were hesitant to care for PLHIV and 21.1% reported they themselves were hesitant. In response, one of the two exercises that address fears of workplace HIV acquisition was retained as originally written. The other was dropped due to time considerations.

With respect to fear as a driver of MSM stigma, some discussion groups raised fear that providing services to MSM could lead to accusations of “promoting” or “encouraging” MSM:

*R1: Honestly when any MSM walks to you, you are eager within to try to get the person out of it [Being MSM]. Though for the first time you will not tell the person, you don't tell it to their face…You are tempted to do that though it's not professional, you are tempted to do it so [otherwise] we become like you are encouraging them to come in and come in, that one it will [be on] our conscience. (FGD, Clinical HCW)*

Perhaps reflecting these fears, 35.1% of respondents reported that they thought their co‐workers were hesitant to care for MSM, while 25.3% reported they themselves were. When asked whether, if they had a choice, they would prefer not to provide services to MSM, 14.1% reported they would prefer not to, whereas only 1.5% indicated they would prefer not to care for PLHIV.

Multiple exercises work in different ways to address these concerns by (1) helping HCWs understand that providing services to MSM does not “promote” MSM or encourage “more” same‐sex behaviour and (2) building skills and confidence to challenge MSM stigma when it occurs, as well as respond to accusations that by providing stigma‐free services or even for attending the workshop, HCWs are promoting MSM. This was done by adapting existing exercises to include new case studies (see confidentiality exercise) and role plays (Be the Change! exercise), as well as adding a new values clarification exercise (see attitudes section).

#### 3.1.3 Driver 3: Attitudes

While there were many stigmatizing attitudes expressed in the qualitative data and confirmed in the survey data (Table 3), two of the most common were: (1) The belief that MSM are “demonic,” “evil,” “morally and religiously wrong” and “sinful,” and (2) The belief that MSM are wilfully “choosing” to engage in sexual behaviour that is “sinful” and “un‐Ghanaian.”

**Table 3:** Attitudes towards HIV and MSM among healthcare facility staff (*N* = 200)^a^

Attitude	Response	PLHIV	MSM
I would feel ashamed if someone in my family was…	Agree	15.3% (*N* = 196)	67.0% (*N* = 194)
	Neither agree nor disagree	6.6% (*N* = 196)	15.5% (*N* = 194)
	Disagree	78.1% (*N* = 196)	17.5% (*N* = 194)
I would feel that I had failed as a parent if I learned that my son was…	Agree	26.2% (*N* = 195)	74.2% (*N* = 194)
	Neither agree nor disagree	14.9% (*N* = 195)	13.9% (*N* = 194)
	Disagree	59.0% (*N* = 195)	11.9% (*N* = 194)
…threaten many of our basic social institutions	Agree	16.7% (*N* = 198)	58.6% (*N* = 198)
	Neither agree nor disagree	16.7% (*N* = 198)	17.7% (*N* = 198)
	Disagree	66.7% (*N* = 198)	23.7% (*N* = 198)
… persons are sinful	Agree	3.6% (*N* = 194)	85.2% (*N* = 196)
	Neither agree nor disagree	9.2% (*N* = 194)	11.2% (*N* = 196)
	Disagree	87.2% (*N* = 194)	3.6% (*N* = 196)
Total: % agreeing with at least one stigmatizing attitude		41.7% (*N* = 199)	92.5% (*N* = 199)

^a^ *N*’s may vary due to non‐response.

Abbreviations: MSM, men who have sex with men; PLHIV, people living with HIV.

These attitudes manifested in a range of verbal and non‐verbal behaviours described in both the HCW and MSM data, like scolding, asking medically unnecessary intrusive questions, bringing up religion to condemn MSM clients or other subtle yet punitive measures:

*They treat you different. Like in my case, after telling her my situation and she [was] asking about my partner and I told her who my partner is, she brought out the Bible. I am a bold person, so when she brought the Bible out, I told her that I wanted to use the washroom. Then I went to another place, but what if I am like other people who are not as bold as I am and because they have had this encounter, decide not to go to any other health facility again? (FGD, MSM)*

The quantitative data reflect many of the stigmatizing attitudes present in the qualitative data (Table 3) and underscore how commonly these beliefs are held. Only 3.6% of HCWs disagreed with the statement that MSM are sinful, compared to 87.2% who disagreed that PLHIV are sinful. Only 16.3% disagreed with the statement that *Being MSM is a behavior that is chosen* (data now shown).

All curriculum exercises address the aspects of stigmatizing attitudes, whether through creating awareness of how stigmatizing attitudes can manifest unconsciously in service delivery (picture exercise) or tackling attitudes directly (new values clarification exercise), building knowledge and understanding of gender and sexual diversity, or through self‐reflection. The panel discussion also allows HCWs to hear first‐hand how stigmatizing attitudes and behaviours hurt and harm MSM clients and puts a “human” face to MSM, building bridges between MSM clients and HCWs.

#### 3.1.4 Driver 4: Facility environment

Breaches of confidentiality of HIV status or being MSM emerged as the one relevant theme under facility environment. Such breaches resulted from HCWs gossiping and not maintaining confidentiality, as well from the way services were structured and physical layout of the facility. Having MSM‐specific services provided in a particular location or at a specific time can disclose that clients are MSM, while the HCF structure/architecture can cause involuntary HIV status disclosure if HIV treatment is provided only on specific days or a specific location:

*People don't trust the confidentiality of the service providers. That's why most guys don't want to go to the facility. They always have the impression that people will gossip about them if they should visit the facility. (FGD, MSM)*

*There was giggling from the time he took the folder and …, people were calling others to come and have a look at the person in question because it looks strange to all of us. So, we could tell that the person knew that we were giggling…though no one rudely spoke, but I think there was a bit of discomfort. (FGD, Clinical HCW)*

The quantitative data show that fears of breaches of confidentiality among PLHIV or MSM are not unfounded. Reporting on the past 6 months, 19% of HCW survey respondents reported observing an HCW disclosing a client's HIV status without their consent, while 9% reported observing disclosure that a client was MSM (Table 4). In response, we maintained and expanded the toolkit exercise on confidentiality by developing and incorporating case studies with MSM‐specific scenarios based on the formative research.

**Table 4:** Observed stigma by healthcare facility staff within their health facilities in the past 6 months (*N* = 200)^a^

In the past 6 months, how often have you observed health care workers, at least once…	PLHIV	MSM
Being unwilling to care for…	16.2% (*N* = 197)	11.1% (*N* = 199)
Providing poorer quality of care to…	13.7% (*N* = 197)	12.6% (*N* = 199)
Talking badly about…	29.5% (*N* = 197)	33.7% (*N* = 199)
Disclosing patient information without consent when not medically necessary	19.0% (*N* = 197)	9.0% (*N* = 199)

^a^ *N*’s may vary due to non‐response.

Abbreviations: MSM, men who have sex with men; PLHIV, people living with HIV.

The survey data provide more insights into potential areas under this driver to address, both through the curriculum and additional intervention activities (Table 5). While 78.5% of HCW respondents indicated they could list several ways to reduce stigma against PLHIV in their HCF, this dropped to 48.7% when asked about addressing MSM stigma. When asked if their facilities had policies to protect PLHIV from discrimination, 78.7% of HCWs agreed, but only 49.7% agreed they had policies to protect MSM.

**Table 5:** Health facility environment stigma factors among healthcare facility staff (*N* = 200)^a^

Statement	Response	PLHIV	MSM
I would feel comfortable working closely with a person who is…	Agree	74.7% (*N* = 194)	37.0% (*N* = 196)
	Neither agree nor disagree	10.3% (*N* = 194)	18.6% (*N* = 196)
	Disagree	14.9% (*N* = 194)	44.3% (*N* = 196)
I will get into trouble at work if I discriminate against …	Agree	77.4% (*N* = 199)	67.8% (*N* = 199)
	Neither agree nor disagree	5.0% (*N* = 199)	10.6% (*N* = 199)
	Disagree	17.6% (*N* = 199)	21.6% (*N* = 199)
My health facility has policies to protect … from discrimination	Agree	78.7% (*N* = 197)	49.7% (*N* = 197)
	Neither agree nor disagree	7.1% (*N* = 197)	23.9% (*N* = 197)
	Disagree	14.2% (*N* = 197)	26.4% (*N* = 197)
I can list several ways I could take action to reduce stigma and discrimination against … in my health facility	True	78.5% (*N* = 195)	48.7% (*N* = 195)
	False	6.7% (*N* = 195)	23.6% (*N* = 195)
	Don't know	14.9% (*N* = 195)	27.7% (*N* = 195)
I am confident that I can challenge stigma and discrimination against MSM in my health facility	True		44.9% (*N* = 198)
	False		29.3% (*N* = 198)
	Don't know		25.8% (*N* = 198)
I am aware of institutional barriers that may inhibit MSM from using health care services	True		27.9% (*N* = 197)
	False		36.5% (*N* = 197)
	Don't know		35.5% (*N* = 197)
I would feel unprepared talking with a MSM client about topics related to their sexuality	True		37.2% (*N* = 196)
	False		49.0% (*N* = 196)
	Don't know		13.8% (*N* = 196)
Health facility policies prevent me from providing quality care to MSM	True		6.1% (*N* = 197)
	False		84.3% (*N* = 197)
	Don't know		9.6% (*N* = 197)
National policies prevent me from providing quality care to MSM	True		7.7% (*N* = 196)
	False		77.0% (*N* = 196)
	Don't know		15.3% (*N* = 196)

^a^ *N*’s may vary due to non‐response.

Abbreviations: MSM, men who have sex with men; PLHIV, people living with HIV.

The training ends with a set of exercises that focus on building skills to challenge stigma in the facility environment and plan for action to reduce stigma. For example, to build skills to challenge stigma, we expanded an assertiveness and role‐playing exercise by adding role‐plays focused on MSM stigma.

## 4 DISCUSSION

Stigma‐reduction interventions need to target the attitudes, beliefs, practices and policies that drive stigma as a means to support engagement across the HIV care continuum [30]. However, research on HCF stigma‐reduction intervention development, adaptation and evaluation does not always explicitly provide this level of detail [22]. The original “Total Facility” curriculum did exactly that by (1) raising awareness on how HIV stigma manifests, (2) addressing fears of HIV transmission through education on routes of transmission, (3) providing a safe, non‐judgemental space for participants to confront the judging, shaming, blaming and stereotyping involved in the stigmatization process and (4) focusing on policies and practices that encourage a stigma‐free HCF environment. The process used in adapting this curriculum specifically sought to understand and document the drivers of intersectional stigma faced by MSM in Ghana in HCFs such that the exercises and activities could be tailored to target these specific drivers.

There is a dearth of interventions that address intersectional stigma, particularly for MSM in low‐resource settings, and support HIV prevention and treatment [35]. The adapted curriculum is novel in that it addresses the intersection of HIV, same‐sex and gender non‐conformity stigmas. The need to recognize and understand an individual's membership in multiple stigmatized groups is a relatively recent phenomena in the field of stigma reduction [8, 57]. While global research highlights the deleterious impacts of stigma on health outcomes [53, 58, 59, 60], intersectional stigma research is just beginning to elucidate how multiple stigmatized conditions and identities are experienced and how their interlocking, compounding effects hamper healthcare access and worsen health outcomes [8]. The overlap of certain stigmatized health conditions and identities—particularly for MSM who shoulder a high burden of HIV—and the rootedness of stigma in larger systems of inequality and power are necessitating researchers and programme managers to consider novel ways to understand and address intersectional stigma [61]. Ultimately, as interventions often take a siloed approach to stigma reduction, addressing only one type of stigma at a single, socio‐ecological level [58], future interventions will need to draw on an intersectional perspective to understand and address the co‐experience of multiple stigmas, marginalization and resilience [8, 62].

Recent decades have witnessed the proliferation of evidence for how to reduce HIV stigma, particularly at the HCF level [22, 31, 32, 63, 64]. As such, there is a rich evidence base around stigma measurement and reduction that provides a solid foundation to apply an intersectional lens to existing evidence‐based practices [30]. Our adaptation approach is an example of implementation research, sharing pragmatic insights around how to draw from an existing HIV stigma‐reduction intervention to address intersectional stigma and promote access to HIV care. Researchers and programme managers should employ implementation science methods to guide and evaluate the adaptation and implementation of stigma‐reduction interventions, particularly in low‐resource settings [65]. To bridge the research‐to‐practice gap in the field of stigma‐reduction, studies need to look beyond efficacy to also include a focus on implementation to identify critical barriers and facilitators to the scale‐up of effective interventions.

## 5 CONCLUSIONS

We adapted the HP+ HIV‐focused HCF stigma‐reduction training curriculum to address intersectional stigma faced by MSM in Ghana and ultimately support HIV prevention and treatment. The research team used a mixed‐methods approach that drew on both formative qualitative data and baseline survey data to understand and document the drivers and manifestations of the intersecting stigma faced by MSM in HCFs—namely, HIV, same‐sex and gender non‐conformity stigmas. The research team used these data to expand or generate new exercises to ensure the curriculum adequately addressed the key drivers of intersectional stigma, including lack of awareness, fear, attitudes and the facility environment. A similar process could serve as a guide for other research and programming efforts seeking to expand existing evidence‐based interventions to address intersectional stigma, particularly for KPs, in low‐resource settings.

## COMPETING INTERESTS

The authors declare that they have no competing interests.

## AUTHORS’ CONTRIBUTIONS

LN, LEN and KT conceived of the parent study. LN and MAS drafted this article with input from KS, DD and GMRA. LN, SC, MC, EM, RV and EG adapted the training curriculum. EM, RV, EG and RA collected the baseline quantitative data. RV oversaw data entry, cleaning and analysis. MAS led qualitative data analysis. MAS, DT, GMRA, KS, SP and DD coded the qualitative data. KT, LN, LEN, EG, FB and PA oversaw the study activities.

## FUNDING

This study is sponsored by the National Institute of Nursing Research, grant number R01 NR019009, which provides the direct financial support for the research. The study is also made possible through core services and support from the Yale Center for Interdisciplinary Research AIDS (P30 MH06224), which provides ongoing consultation on research design and methods, including on methods to facilitate continuity of HIV/AIDS research operations during COVID‐19 pandemic. The Yale AIDS Prevention Training program (T32 MH020031) provides postdoctoral trainees to assist with research project coordination.

## Data Availability

The datasets generated and analyzed during the current study are not publicly available due to the high risk of persecution and severe adverse social consequences related to the socio‐political sensitivity of the topic of same‐sex behaviors in Ghana; however, data are available from the corresponding author on reasonable request.

## References

[1] UN General Assembly . Political declaration on HIV and AIDS: ending inequalities and getting on track to end AIDS by 2030. 74th Plenary Meeting 8 June 2021, Switzerland; 2021.

[2] UNAIDS . Prevailing against pandemics by putting people at the centre. World AIDS Day Report. Geneva: UNAIDS; 2020.

[3] Nyblade L , Mingkwan P , Stockton MA . Stigma reduction: an essential ingredient to ending AIDS by 2030. Lancet HIV. 2021;8(2):e106–13. 33539757 10.1016/S2352-3018(20)30309-X

[4] Logie CH , Newman PA , Chakrapani V , Shunmugam M . Adapting the minority stress model: associations between gender non‐conformity stigma, HIV‐related stigma and depression among men who have sex with men in South India. Soc Sci Med. 2012;74(8):1261–8. 22401646 10.1016/j.socscimed.2012.01.008

[5] Philbin MM , Hirsch JS , Wilson PA , Ly AT , Giang LM , Parker RG . Structural barriers to HIV prevention among men who have sex with men (MSM) in Vietnam: diversity, stigma, and healthcare access. PLoS One. 2018;13(4):e0195000. 29614104 10.1371/journal.pone.0195000 PMC5882136

[6] Nybalde L. , Addo NA , Mingkwan P , Vormawor R , Stewart C , Gyamera E , et al. Understanding and responding to stigma and discrimination in health facilities in Ghana: intervention endline report. Washington, DC: Palladium, Health Policy Plus; 2018.

[7] Bowleg L . The problem with the phrase women and minorities: intersectionality—an important theoretical framework for public health. Am J Public Health. 2012;102:1267–73. 22594719 10.2105/AJPH.2012.300750 PMC3477987

[8] Turan JM , Elafros MA , Logie CH , Banik S , Turan B , Crockett KB , et al. Challenges and opportunities in examining and addressing intersectional stigma and health. BMC Med. 2019;17(1):1–15. 30764816 10.1186/s12916-018-1246-9 PMC6376691

[9] Abubakari GMR , Dada D , Nur J , Turner D , Otchere A , Tanis L , et al. Intersectional stigma and its impact on HIV prevention and care among MSM and WSW in sub‐Saharan African countries: a protocol for a scoping review. BMJ Open. 2021;11(8):e047280. 10.1136/bmjopen-2020-047280 PMC8351482 34362801

[10] Turan JM , Elafros MA , Logie CH , Banik S , Turan B , Crockett KB , et al. Challenges and opportunities in examining and addressing intersectional stigma and health. BMC Med. 2019;17(1):7. 30764816 10.1186/s12916-018-1246-9 PMC6376691

[11] Pachankis JE , Hatzenbuehler ML , Berg RC , Fernández‐Dávila P , Mirandola M , Marcus U , et al. Anti‐LGBT and anti‐immigrant structural stigma: an intersectional analysis of sexual minority men's HIV risk when migrating to or within Europe. J Acquir Immune Defic Syndr. 2017;76(4):356. 28787329 10.1097/QAI.0000000000001519 PMC5659919

[12] Pachankis JE , Hatzenbuehler ML , Hickson F , Weatherburn P , Berg RC , Marcus U , et al. Hidden from health: structural stigma, sexual orientation concealment, and HIV across 38 countries in the European MSM Internet Survey. AIDS. 2015;29(10):1239. 26035323 10.1097/QAD.0000000000000724 PMC4820755

[13] Flores AR . Social acceptance of LGBT people in 174 countries 1981 to 2017. Los Angelos, CA: UCLA School of Law Williams Institute; 2019.

[14] Stahlman S , Sanchez TH , Sullivan PS , Ketende S , Lyons C , Charurat ME , et al. The prevalence of sexual behavior stigma affecting gay men and other men who have sex with men across sub‐Saharan Africa and in the United States. JMIR Public Health Surveill. 2016;2(2):e5824. 10.2196/publichealth.5824 PMC4978863 27460627

[15] Schwartz SR , Nowak RG , Orazulike I , Keshinro B , Ake J , Kennedy S , et al. The immediate effect of the Same‐Sex Marriage Prohibition Act on stigma, discrimination, and engagement on HIV prevention and treatment services in men who have sex with men in Nigeria: analysis of prospective data from the TRUST cohort. Lancet HIV. 2015;2(7):e299–306. 26125047 10.1016/S2352-3018(15)00078-8 PMC4481876

[16] Kushwaha S , Lalani Y , Maina G , Ogunbajo A , Wilton L , Agyarko‐Poku T , et al. “But the moment they find out that you are MSM…”: a qualitative investigation of HIV prevention experiences among men who have sex with men (MSM) in Ghana's health care system. BMC Public Health. 2017;17(1):770. 28974257 10.1186/s12889-017-4799-1 PMC5627492

[17] Atuguba RA . Homosexuality in Ghana: morality, law, human rights. J Pol Law. 2019;12:113.

[18] Ogunbajo A , Kershaw T , Kushwaha S , Boakye F , Wallace‐Atiapah N‐D , Nelson LE . Barriers, motivators, and facilitators to engagement in HIV care among HIV‐infected Ghanaian men who have sex with men (MSM). AIDS Behav. 2018;22(3):829–39. 28550380 10.1007/s10461-017-1806-6 PMC5995561

[19] Mendos LR , Botha K , Lelis RC , de la Peña EL , Savelev I , Tan D . State‐sponsored homophobia 2020: global legislation overview update. Geneva: ILGA; 2020.

[20] Semugoma P , Beyrer C , Baral S . Assessing the effects of anti‐homosexuality legislation in Uganda on HIV prevention, treatment, and care services. SAHARA J. 2012;9(3):173–6. 23237074 10.1080/17290376.2012.744177

[21] Smith MK , Xu RH , Hunt SL , Wei C , Tucker JD , Tang W , et al. Combating HIV stigma in low‐and middle‐income healthcare settings: a scoping review. J Int AIDS Soc. 2020;23(8):e25553. 32844580 10.1002/jia2.25553 PMC7448195

[22] Nyblade L , Stockton MA , Giger K , Bond V , Ekstrand ML , Mc Lean R , et al. Stigma in health facilities: why it matters and how we can change it. BMC Med. 2019;17(1):1–15. 30764806 10.1186/s12916-019-1256-2 PMC6376713

[23] Gu LY , Zhang N , Mayer KH , McMahon JM , Nam S , Conserve DF , et al. Autonomy‐supportive healthcare climate and HIV‐related stigma predict linkage to HIV care in men who have sex with men in Ghana, West Africa. J Int Assoc Provid AIDS Care. 2021;20:2325958220978113. 33733909 10.1177/2325958220978113 PMC7983411

[24] Nyblade L , Stangl A , Weiss E , Ashburn K . Combating HIV stigma in health care settings: what works? J Int AIDS Soc. 2009;12(1):1–7. 10.1186/1758-2652-12-15 PMC2731724 19660113

[25] UNAIDS . Global partnership for action to eliminate all forms of HIV‐related stigma and discrimination. Geneva: Joint United Nations Programme on HIV/AIDS; 2018.

[26] UNAIDS . Global agenda for zero discrimination in health‐care settings. Geneva: UNAIDS; 2017.

[27] Fast ‐Track Cities Initiative . About fast track cities: International Association of Providers of AIDS Care. 2021. Available from: https://www.fast‐trackcities.org/about. Accessed 27 July, 2021.

[28] Feyissa GT , Lockwood C , Woldie M , Munn Z . Reducing HIV‐related stigma and discrimination in healthcare settings: a systematic review of guidelines, tools, standards of practice, best practices, consensus statements and systematic reviews. J Multidiscip Healthcare. 2018;11:405–16. 10.2147/JMDH.S170720 PMC6118284 30214222

[29] Nyblade L , Mbuya‐Brown RJ , Ezekiel MJ , Addo NA , Sabasaba AN , Atuahene K , et al. A total facility approach to reducing HIV stigma in health facilities: implementation process and lessons learned. AIDS. 2020;34:S93–102. 32881798 10.1097/QAD.0000000000002585

[30] Nyblade L , Mingkwan P , Stockton MA . Stigma reduction: an essential ingredient to reaching the 95‐95‐95 targets by 2030. Lancet HIV. 2021;8(2):e106–13. 33539757 10.1016/S2352-3018(20)30309-X

[31] Sengupta S , Banks B , Jonas D , Miles MS , Smith GC . HIV interventions to reduce HIV/AIDS stigma: a systematic review. AIDS Behav. 2011;15(6):1075–87. 21088989 10.1007/s10461-010-9847-0 PMC3128169

[32] Stangl AL , Lloyd JK , Brady LM , Holland CE , Baral S . A systematic review of interventions to reduce HIV‐related stigma and discrimination from 2002 to 2013: how far have we come? J Int AIDS Soc. 2013;16(3 Suppl 2):18734. 24242268 10.7448/IAS.16.3.18734 PMC3833106

[33] van der Elst EM , Kombo B , Gichuru E , Omar A , Musyoki H , Graham SM , et al. The green shoots of a novel training programme: progress and identified key actions to providing services to MSM at Kenyan health facilities. J Int AIDS Soc. 2015;18(1):20226. 26493863 10.7448/IAS.18.1.20226 PMC4615801

[34] Van der Elst EM , Smith AD , Gichuru E , Wahome E , Musyoki H , Muraguri N , et al. Men who have sex with men sensitivity training reduces homoprejudice and increases knowledge among Kenyan healthcare providers in coastal Kenya. J Int AIDS Soc. 2013;16:18748. 24321111 10.7448/IAS.16.4.18748 PMC3852129

[35] Dunbar W , Labat A , Raccurt C , Sohler N , Pape JW , Maulet N , et al. A realist systematic review of stigma reduction interventions for HIV prevention and care continuum outcomes among men who have sex with men. Int J STD AIDS. 2020;31(8):712–23. 32631213 10.1177/0956462420924984

[36] Lyons CE , Ketende S , Diouf D , Drame FM , Liestman B , Coly K , et al. Potential impact of integrated stigma mitigation interventions in improving HIV/AIDS service delivery and uptake for key populations in Senegal. J Acquir Immune Defic Syndr. 2017;74: Suppl 1, S52–9. 27930612 10.1097/QAI.0000000000001209 PMC5147043

[37] Nelson LE , Nyblade L , Torpey K , Logie C , Qian H‐Z , Manu A , et al. Multi‐level intersectional stigma reduction intervention to increase HIV testing among men who have sex with men in Ghana: a cluster randomized controlled trial protocol. PLoS One. 2021;16(11):e0259324. 34843529 10.1371/journal.pone.0259324 PMC8629287

[38] Placek CD , Nishimura H , Hudanick N , Stephens D , Madhivanan P . Reframing HIV stigma and fear. Hum Nat. 2019;30(1):1–22. 30661161 10.1007/s12110-018-09335-z PMC6446936

[39] Stokols D . Establishing and maintaining healthy environments: toward a social ecology of health promotion. Am Psychol. 1992;47(1):6. 1539925 10.1037//0003-066x.47.1.6

[40] Wingood GM , DiClemente RJ . The ADAPT‐ITT model: a novel method of adapting evidence‐based HIV interventions. J Acquir Immune Defic Syndr. 2008;47:40–6. 10.1097/QAI.0b013e3181605df1 18301133

[41] Abubakari GMR , Nelson LE , Ogunbajo A , Boakye F , Appiah P , Odhiambo A , et al. Implementation and evaluation of a culturally grounded group‐based HIV prevention programme for men who have sex with men in Ghana. Glob Public Health. 2021;16(7):1028–45. 33050773 10.1080/17441692.2020.1832555 PMC8728790

[42] Abubakari GM , Owusu‐Dampare F , Ogunbajo A , Gyasi J , Adu M , Appiah P , et al. HIV Education, Empathy, and Empowerment (HIVE [3]): a peer support intervention for reducing intersectional stigma as a barrier to HIV testing among men who have sex with men in Ghana. Int J Environ Res Public Health. 2021;18(24):13103. 34948712 10.3390/ijerph182413103 PMC8702001

[43] Kidd RSC , Stockton M , Nyblade L . Facilitator's training guide for a stigma‐free health facility. Futures Group: Health Policy Project; 2015.

[44] Clay S , Chonta M , Chiiya C , Stewart C , Nyblade L . Towards stigma‐free health facilities in Ghana: guide for trainers. Washington, DC: Palladium, Health Policy Plus; 2017.

[45] Nyblade L , Stewart C , Kiwia P , Manyama W , Mbuya‐Brown R , Bowsky S . Towards stigma‐free health facilities in Tanzania: guide for trainers. Palladium, Health Policy Plus; 2018.

[46] Mbuya‐Brown R , Mangi E , Addo NA , Sabasaba AN , Atuahene K , Kraemer JD . A total facility approach to reducing HIV stigma in health facilities: implementation process and lessons learned. AIDS. 2020;34:S93–102. 32881798 10.1097/QAD.0000000000002585

[47] Siraprapasiri T , Srithanaviboonchai K , Chantcharas P , Suwanphatthana N , Ongwandee S , Khemngern P , et al. Integration and scale‐up of efforts to measure and reduce HIV‐related stigma: the experience of Thailand. AIDS. 2020;34:S103–4. 32881799 10.1097/QAD.0000000000002586

[48] Nyblade L , Addo NA , Atuahene K , Alsoufi N , Gyamera E , Jacinthe S , et al. Results from a difference‐in‐differences evaluation of health facility HIV and key population stigma‐reduction interventions in Ghana. J Int AIDS Soc. 2020;23:e25483. 32329153 10.1002/jia2.25483 PMC7180216

[49] Johnson TP . Snowball sampling: introduction. Wiley StatsRef: Statistics Reference Online; 2014.

[50] Saldaña J . Chapter 2: Writing analytic memos about narrative and visual data. In Jai Seaman (ed). The coding manual for qualitative researchers. Sage Publications; 2016. pp.43–55.

[51] Braun V , Clarke V . Using thematic analysis in psychology. Qual Res Psychol. 2006;3(2):77–101.

[52] Attride‐Stirling J . Thematic networks: an analytic tool for qualitative research. Qual Res. 2001;1(3):385–405.

[53] Stangl AL , Earnshaw VA , Logie CH , van Brakel W , Simbayi LC , Barré I , et al. The health stigma and discrimination framework: a global, crosscutting framework to inform research, intervention development, and policy on health‐related stigmas. BMC Med. 2019;17(1):1–13. 30764826 10.1186/s12916-019-1271-3 PMC6376797

[54] Damschroder LJ , Reardon CM , Lowery JC . The Consolidated Framework for Implementation Research (CFIR). In Handbook on implementation science. Edward Elgar Publishing; 2020.

[55] Nyblade L , Mbuya‐Brown RJ , Ezekiel MJ , Addo NA , Sabasaba AN , Atuahene K , et al. A total facility approach to reducing HIV stigma in health facilities: implementation process and lessons learned. AIDS; 2020;34: Suppl 1, S93–102. 10.1097/QAD.0000000000002585 32881798

[56] International HIV/AIDS Alliance . Keep the best, change the rest. Participatory tools for working with communities on gender and sexuality. Brighton: International HIV/AIDS Alliance; 2007.

[57] Henkel KE , Brown K , Kalichman SC . AIDS‐related stigma in individuals with other stigmatized identities in the USA: a review of layered stigmas. Soc Personal Psychol Compass. 2008;2(4):1586–99.

[58] Van Brakel WH , Cataldo J , Grover S , Kohrt BA , Nyblade L , Stockton M , et al. Out of the silos: identifying cross‐cutting features of health‐related stigma to advance measurement and intervention. BMC Med. 2019;17(1):1–17. 30764817 10.1186/s12916-018-1245-x PMC6376667

[59] Kane JC , Elafros MA , Murray SM , Mitchell EM , Augustinavicius JL , Causevic S , et al. A scoping review of health‐related stigma outcomes for high‐burden diseases in low‐ and middle‐income countries. BMC Med. 2019;17(1):1–40. 30764819 10.1186/s12916-019-1250-8 PMC6376728

[60] Hatzenbuehler ML , Phelan JC , Link BG . Stigma as a fundamental cause of population health inequalities. Am J Public Health. 2013;103(5):813–21. 23488505 10.2105/AJPH.2012.301069 PMC3682466

[61] Jackson‐Best F , Edwards N . Stigma and intersectionality: a systematic review of systematic reviews across HIV/AIDS, mental illness, and physical disability. BMC Public Health. 2018;18(1):1–19. 10.1186/s12889-018-5861-3 PMC6062983 30049270

[62] Sangaramoorthy T , Jamison AM , Dyer TV . HIV stigma, retention in care, and adherence among older black women living with HIV. J Assoc Nurses AIDS Care. 2017;28(4):518–31. 28366556 10.1016/j.jana.2017.03.003

[63] Joint United Nations Programme on HIV/AIDS . Evidence for eliminating HIV‐related stigma and discrimination—guidance for countries to implement effective programmes to eliminate HIV‐related stigma and discrimination in six settings. Geneva: UNAIDS; 2020.

[64] Andersson GZ , Reinius M , Eriksson LE , Svedhem V , Esfahani FM , Deuba K , et al. Stigma reduction interventions in people living with HIV to improve health‐related quality of life. Lancet HIV. 2020;7(2):e129–40. 31776098 10.1016/S2352-3018(19)30343-1 PMC7343253

[65] Kemp CG , Jarrett BA , Kwon C‐S , Song L , Jetté N , Sapag JC , et al. Implementation science and stigma reduction interventions in low‐ and middle‐income countries: a systematic review. BMC Med. 2019;17(1):1–18. 30764820 10.1186/s12916-018-1237-x PMC6376798

