# Corrigendum

**DOI:** 10.1002/jia2.26015

**Published:** 2022-09-25

In the article [1], there were errors in the article title and Figure 1 on page 87.

In the title, the word ‘intervention’ was missing and should be corrected.

The title reads: Using a mixed‐methods approach to adapt an HIV stigma reduction to address intersectional stigma faced by men who have sex with men in Ghana

The title should read: Using a mixed‐methods approach to adapt an HIV stigma reduction **intervention** to address intersectional stigma faced by men who have sex with men in Ghana

In Figure 1 flowchart and text, the MSM IDIs number should be changed from 8 IDIs to 10 IDIs. The correct Figure 1 is shown below.

**Figure 1:**
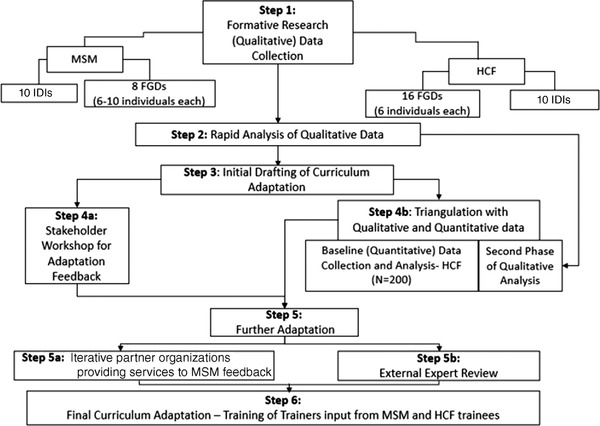
Curriculum adaptation methods and process. Abbreviations: FGDs, focus group discussions; HCF, health care facilities; IDIs, in‐depth interviews; MSM, men who have sex with men.

The online version of the article was updated.
